# A mobile element-based evolutionary history of guenons (tribe Cercopithecini)

**DOI:** 10.1186/1741-7007-5-5

**Published:** 2007-01-31

**Authors:** Jinchuan Xing, Hui Wang, Yuhua Zhang, David A Ray, Anthony J Tosi, Todd R Disotell, Mark A Batzer

**Affiliations:** 1 Department of Biological Sciences, Biological Computation and Visualization Center, Center for Bio-Modular Multi-scale Systems, Louisiana State University, Baton Rouge, LA 70803, USA; 2 Department of Biology, West Virginia University, PO Box 6057, Morgantown, West VA 26506, USA; 3 Department of Anthropology, New York University, New York, NY 10003, USA; 4 Department of Human Genetics, University of Utah Health Sciences Center, Salt Lake City, UT 84112, USA

## Abstract

**Background:**

Guenons (tribe Cercopithecini) are a species-rich group of primates that have attracted considerable attention from both primatologists and evolutionary biologists. The complex speciation pattern has made the elucidation of their relationships a challenging task, and many questions remain unanswered. SINEs are a class of non-autonomous mobile elements and are essentially homoplasy-free characters with known ancestral states, making them useful genetic markers for phylogenetic studies.

**Results:**

We identified 151 novel *Alu *insertion loci from 11 species of tribe Cercopithecini, and used these insertions and 17 previously reported loci to infer a phylogenetic tree of the tribe Cercopithecini. Our results robustly supported the following relationships: (i) *Allenopithecus *is the basal lineage within the tribe; (ii) *Cercopithecus lhoesti *(L'Hoest's monkey) forms a clade with *Chlorocebus aethiops *(African green monkey) and *Erythrocebus patas *(patas monkey), supporting a single arboreal to terrestrial transition within the tribe; (iii) all of the *Cercopithecus *except *C. lhoesti *form a monophyletic group; and (iv) contrary to the common belief that *Miopithecus *is one of the most basal lineages in the tribe, *M. talapoin *(talapoin) forms a clade with arboreal members of *Cercopithecus*, and the terrestrial group (*C. lhoesti*, *Chlorocebus aethiops *and *E. patas*) diverged from this clade after the divergence of Allenopithecus. Some incongruent loci were found among the relationships within the arboreal *Cercopithecus *group. Several factors, including incomplete lineage sorting, concurrent polymorphism and hybridization between species may have contributed to the incongruence.

**Conclusion:**

This study presents one of the most robust phylogenetic hypotheses for the tribe Cercopithecini and demonstrates the advantages of SINE insertions for phylogenetic studies.

## Background

Guenons (tribe Cercopithecini) are a species-rich group of primates with a distribution throughout sub Saharan Africa. With their diverse morphology, ecology, behavior and social organizations, guenons have attracted considerable attention from both primatologists and evolutionary biologists [1,2]. In addition, some species in the tribe (e.g. *Chlorocebus aethiops*) have been widely used in biomedical studies [3-5]. Based on Groves' classification [6], the tribe Cercopithecini consists of five genera (*Erythrocebus, Chlorocebus, Cercopithecus, Miopithecus *and *Allenopithecus*) comprising 36 species. The evolutionary history of guenons may include a rapid basal radiation, and it is very likely that speciation is an ongoing process within the tribe [1,7]. This complex speciation pattern has made the elucidation of the species relationships a challenging task, and many questions remain unanswered about the tribe composition (e.g. inclusion of *Allenopithecus *in the tribe), genera definitions (e.g. whether to consider *Chlorocebus *as a separate genus) and in particular, the phylogenetic relationships among genera and species [6,8,9].

To elucidate the phylogeny of tribe Cercopithecini, several molecular studies have been conducted, and some aspects of their phylogeny have been inferred with a degree of confidence [7,10-14]. Nevertheless, two major questions remain unanswered:

• (i) is genus *Cercopithecus *paraphyletic and what are the species relationships within the genus?

• (ii) what is the branching order of the major groups?

Regarding the first question, *Cercopithecus *was previously considered to be a monophyletic group [10], but recent molecular [7,13,14] and karyotype studies [11] support grouping of the terrestrial taxa, *Cercopithecus lhoesti *species group, *Chlorocebus *and *Erythrocebus*, into a single lineage. This topology supports a single arboreal to terrestrial transition within the tribe, and divides the current genus *Cercopithecus *into a paraphyletic group. For the phylogenetic relationships among the arboreal members of *Cercopithecus*, several types of molecular data, including X and Y chromosome DNA sequences, protein sequences and karyotypes, recognize a clade containing a *cephus *species group and a *mitis *group. X chromosome DNA and protein studies also support a *neglectus *+ *diana *+ *mona *group clade (see summary in Tosi *et al *[7]). Nevertheless, a robust phylogenetic hypothesis of lower-level relationships among arboreal *Cercopithecus *species is not yet confirmed, as there is discordant evidence [7,9].

The other major remaining question is the relationships between the major groups within Cercopithecini. It is thought that the tribe can be divided into four major clades: an arboreal *Cercopithecus *clade, a terrestrial clade (*C. lhoesti *species group, *Chlorocebus *and *Erythrocebus*), *Miopithecus *and *Allenopithecus*. *Miopithecus *and *Allenopithecus *are usually considered to be the most basal lineages of the tribe [11,15,16]. Although recent studies [7,14] have provided robust support for the most basal lineage position of *Allenopithecus*, the relationships of the other three major clades (i.e. the arboreal *Cercopithecus *clade, the terrestrial clade, and *Miopithecus*) remain unclear. A set of molecular markers that are independent of DNA sequence data may help to resolve these phylogenetic questions in guenons.

*S*hort *in*terspersed *e*lement (SINE) insertions are a class of retrotransposons that integrate into a genome via an RNA intermediate [17]. SINE-based phylogenetic systems were put into practice in the early 1990s [18,19], and certain unique characteristics make them particularly promising for evolutionary analyses [20-23]. Briefly, SINEs are usually unidirectional characters, with the absence of the insertion being the ancestral state. Precise removal of SINEs is extremely rare after their fixation in the genome, and is very unlikely to happen in multiple genomes [24]. In addition, SINE-based analysis does not rely directly on analyzing DNA sequence data, which makes SINEs an independent complement to traditional DNA-based molecular studies that focus on sequence substitutions. In recent years, SINE-based phylogenetic analysis has proven to be a powerful tool for various levels of phylogenetic studies, and many controversial phylogenetic relationships that could not be solved using traditional molecular data have been successfully elucidated [21-23].

*Alu *elements are the most successful SINEs in primate genomes in terms of copy number. During primate evolution, *Alu *elements have proliferated in all primates and expanded to more than one million copies in the human genome [25,26]. Several studies have been conducted to infer primate phylogenetic relationships using *Alu *elements, including the human, chimpanzee and gorilla trichotomy [27], Old World [28] and New World monkey phylogeny [29], tarsier affiliation [30], and strepsirrhine phylogeny [31].

Using a combination of computational data-mining and PCR-based display methods, we identified 168 *Alu *insertion loci, which contained a total of 179 *Alu *insertions that had integrated into 11 cercopithecine genomes at various times. These insertions were used to construct a robustly supported phylogenetic hypothesis for the tribe Cercopithecini. The major relationships are: (i) *Allenopithecus *is the basal lineage of the tribe; (ii) *C. lhoesti *(L'Hoest's monkey) forms a clade with *Chlorocebus aethiops *(African green monkey) and *E. patas *(patas monkey), supporting a single transition to a terrestrial lifestyle; (iii) all *Cercopithecus *species except *C. lhoesti *form a monophyletic group; and (iv) contrary to the usual hypothesis that *Miopithecus *is one of the most primitive lineages in the tribe, our results suggest that the divergence between the arboreal *Cercopithecus *group and *M. talapoin *happened after the divergence of the terrestrial group.

## Results

**Figure 1:**
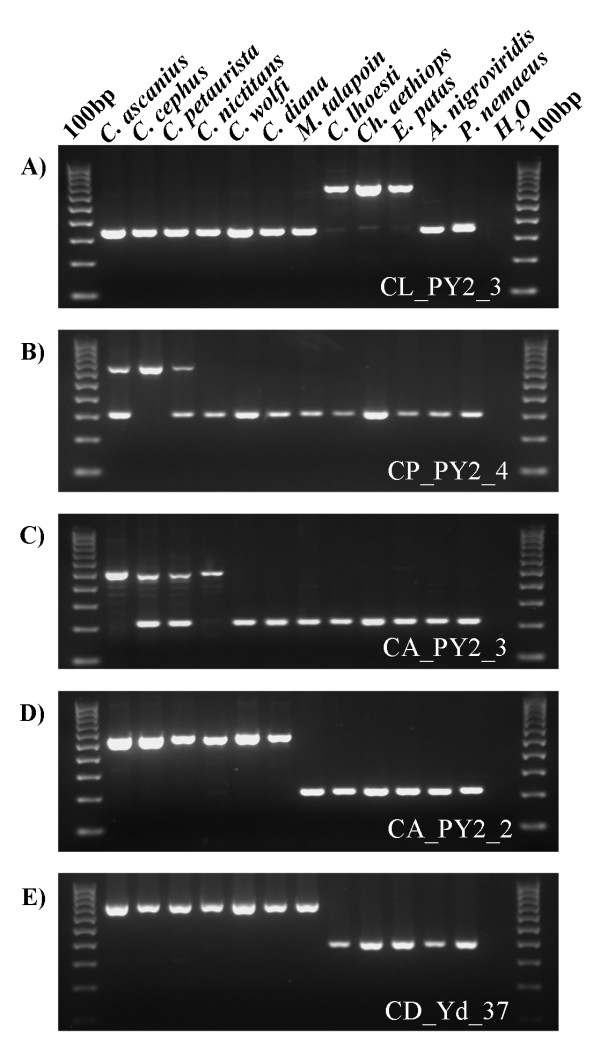
**Phylogenetic analysis of *Alu *insertion polymorphisms in guenon genomes**. Gel electrophoresisresults of five amplifications used to determine the phylogenetic origin of individual *Alu *insertions in Cercopithecini species are shown. Upper DNA fragments indicate "filled" sites where *Alu *elements have inserted, and lower fragments indicate "empty" sites containing no *Alu *insertion. The DNA template used in each reaction and the locus designation are shown. (A) An *Alu *insertion specific for terrestrial guenons (*C. lhoesti*, *Chlorocebus aethiops *and *E. patas*); (B) An *Alu *insertion restricted to the *cephus *species group (*C. ascanius*, *C. cephus *and *C. petaurista*); (C) An *Alu *insertion clustering the *cephus *species group and *C. nictitans*; (D) An *Alu *insertion restricted the arboreal *Cercopithecus *group; (E) An *Alu *element present in the arboreal *Cercopithecus *group and *M. talapoin*.

In total, 168 loci were selected for the phylogenetic analysis. Of these, 144 were identified using PCR display methodology, 7 were collected from available African green monkey (*Chlorocebus aethiops*) genomic sequences using a computational data mining approach (see Methods for details) and 17 loci were selected from a previous study [28]. All loci were genotyped on a primate panel composed of 12 Old World primate species (Table 1), including 11 species within tribe Cercopithecini, with *Pygathrix nemaeus *(red-shanked Douc langur) as the outgroup. Gel electrophoresis results of five amplifications are shown in Figure 1. For the potential informative loci (i.e. loci in which more than one but not all species showed *Alu *insertion-sized amplicons), all of the PCR amplicons that appeared to have *Alu *insertions were sequenced to verify the presence of the *Alu *elements.

**Figure 2:**
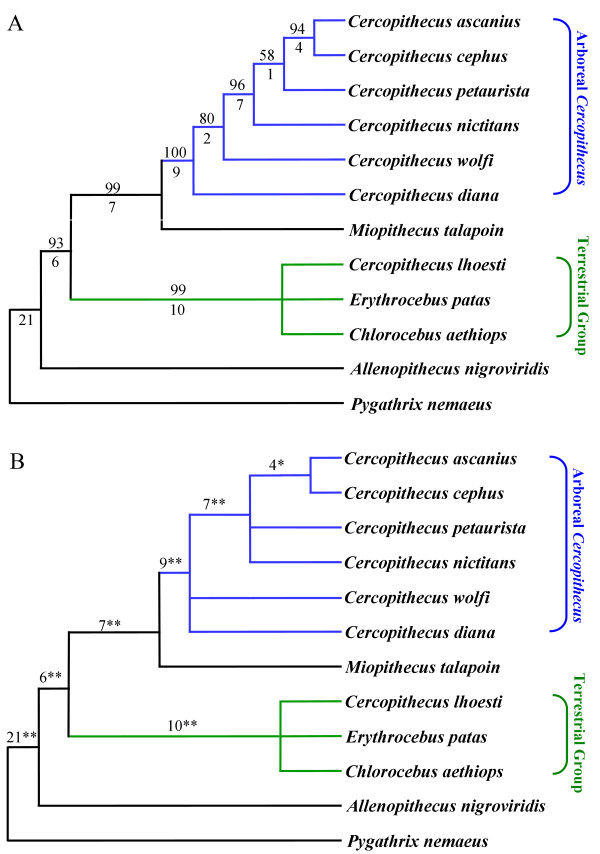
**Cladograms of tribe Cercopithecini phylogenetic relationships derived from *Alu *insertion polymorphisms**. The blue section of each phylogeny indicates species of *Cercopithecus *with an arboreal lifestyle and the green section indicates species with a terrestrial lifestyle. (A) The strict consensus of the two most parsimonious trees from analysis of 179 *Alu *insertion polymorphisms. The numbers above the branches indicate the percentage of bootstrap replicates (10000 iterations) producing trees that include that node. Numbers below the branches indicate the numbers of unambiguous insertions supporting each node. (B) The collapsed version of tree A. Branches that did not gain statistically significant support in the likelihood test [32] were collapsed into polytomies. The significance level of each node is indicated by either * (*p *< 0.05) or ** (*p *< 0.01).

The sequencing verification recovered 11 loci that contained adjacent independent insertion events (i.e. independent insertions in different species that are within sufficiently close genomic proximity to be amplified by a single set of PCR primers; see Discussion for details). These adjacent independent insertion events were treated as independent markers in the subsequent analysis. Overall, 179 markers were used for the phylogenetic analysis. For presence/absence characters in which the absence of the marker is assumed to be the ancestral state, Dollo parsimony is the most appropriate analysis. Therefore, we implemented an exhaustive search using Dollo parsimony, designating *P. nemaeus *as an outgroup taxon. The analysis indicated that 78 loci were parsimony informative and resulted in two most parsimonious trees; the strict consensus for these two trees is shown in Figure 2A (219 steps, consistency index = 0.817; homoplasy index = 0.183; retention index = 0.802). A likelihood test for every branch [32] and 10000 bootstrap replicates were performed. The significance level and the percentage of bootstrap replicates supporting each branch are indicated. A collapsed version of this tree is shown (Figure 2B), in which branches that did not garner statistically significant support based on the likelihood test were collapsed into polytomies.

### The monophyly of the terrestrial group

We identified 10 *Alu *insertions shared by *C. lhoesti*, *Chlorocebus aethiops *and *E. patas*. This result strongly supported (*p *< 0.001) [32] the monophyly of the terrestrial group and the paraphyly of the previously defined genus *Cercopithecus*. This result is congruent with the single transition hypothesis, which states that there was only one transition from an arboreal to terrestrial lifestyle within the tribe [7,14,33]. Unfortunately, we did not identify enough informative markers to gain significant support for the relationships between taxa within the terrestrial group.

### The branching order of the major clades

Given the unambiguous clustering of a single terrestrial clade, our results support the division of the tribe Cercopithecini into four major clades: the arboreal *Cercopithecus *species, a terrestrial clade, *Miopithecus*, and *Allenopithecus*. Six *Alu *insertions were shared by all taxa except *A. nigroviridis*. This unambiguously placed *Allenopithecus *as the basal lineage within the tribe (*p *< 0.01). Another seven insertions joined *M. talapoin *to all arboreal *Cercopithecus *species (i.e. *C. ascanius*, *C. cephus*, *C. petaurista*, *C. wolfi*, *C. diana *and *C. nictitans*). These results suggest that the terrestrial group diverged after *Allenopithecus *but before the separation of *Miopithecus *from the arboreal *Cercopithecus *species (*p *< 0.01). The divergence of *Miopithecus *and the monophyly of the six arboreal *Cercopithecus *species were supported by nine *Alu *insertion loci present in all arboreal taxa but not in *Miopithecus *(*p *< 0.01).

### The relationships among the arboreal *Cercopithecus *species

**Figure 3:**
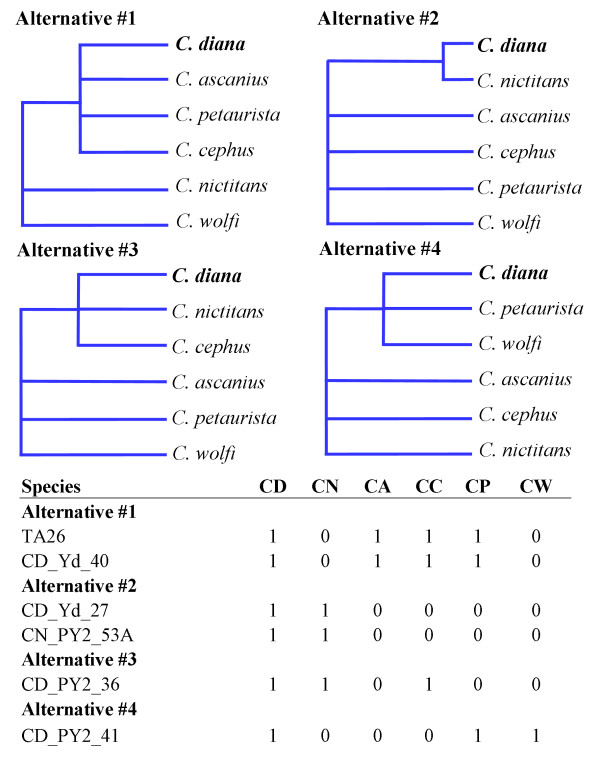
***Alu *insertions support different phylogenetic hypotheses for the affiliation of *C. diana***. Four alternative hypotheses and the markers supporting each hypothesis are shown. Presence of the insert was coded as "1" and absence of the insert as "0". CD, *C. diana*; CN, *C. nictitans*; CP, *C. petaurista*; CA, *C. ascanius*; CC, *C. cephus*; CW, *C. wolfi*.

Of the six arboreal *Cercopithecus *species we tested, two clades were well supported. *C. ascanius *and *C. cephus *were clustered together to the exclusion of *C. petaurista*, based on four *Alu *insertions (*p *< 0.05). The *C. ascanius *and *C. cephus *clade was linked to *C. petaurista *and *C. nictitans *by seven insertions to form a polytomy (*p *< 0.01). Despite the relatively strong support for these two nodes, we should note that there were loci that supported alternative topologies among the arboreal *Cercopithecus *species. For example, when the affiliation of *C. diana *is considered, *Alu *insertions present in *C. diana *suggested at least four alternative phylogenetic hypothesis (Figure 3). Two *Alu *insertions (TA26 and CD_Yd_40) grouped *C. diana *with *C. ascanius*, *C. cephus *and *C. petaurista*, but not *C. nictitans*. In contrast, two other *Alu *elements (CD_Yd_27 and CN_PY2_53A) were found only in *C. diana *and *C. nictitans*. In addition, one marker (CD_PY2_36) supported the grouping of *C. diana*, *C. nictitans *and *C. cephus*, and one marker (CD_PY2_41) grouped *C. diana*, *C. wolfi *and *C. petaurista *together. Owing to this incongruence and the small number of makers supporting each hypothesis, none of these hypotheses gained statistically significant support using the likelihood test.

Another noteworthy result is that a number of *Alu *insertion loci appeared to be heterozygous in certain species (e.g. both a filled band and an empty band are present) (Figures 1B and 1C). Several mechanisms, including incomplete lineage sorting, concurrent polymorphism and introgression may have contributed to this finding.

## Discussion

### The placement of *Allenopithecus *and *Miopithecus*

Our results indicate that *Allenopithecus *is the most basal lineage of the tribe. A monophyletic terrestrial clade comprising *C. lhoesti*, *E. patas*, and *Chlorocebus aethiops *was the next to diverge from other guenons after the split of *Allenopithecus*, and *Miopithecus *subsequently diverged from the rest of arboreal *Cercopithecus*. This placement of *Miopithecus *represents a major difference compared with other phylogenetic analyses of the Cercopithecini. Despite the general view that *Miopithecus *may share some primitive features of the group (e.g. sexual swelling), no studies to date have provided robust support for a definitive placement for *Miopithecus*. For example, in a study using Y-chromosome DNA data [14], a four-way polytomy for *Allenopithecus*; *Miopithecus*, a terrestrial clade and an arboreal *Cercopithecus *clade was obtained. Studies using X-chromosome sequences identified *Allenopithecus *as the most basal lineage of the tribe, although a three-way polytomy consisting of *Miopithecus*, the terrestrial clade and the arboreal *Cercopithecus *clade persisted [7,14]. Our results represent the first statistically significant support for the clustering of *Miopithecus *with the arboreal *Cercopithecus *clade.

### Phylogenetic relationship of the arboreal *Cercopithecus *species

Owing to rapid speciation and the resulting close relationships within the genus *Cercopithecus*, a detailed lower-level hypothesis of relationships has been lacking. To date, the only robustly supported relationships are the separation of a *cephus *group + *mitis *group clade, and a *neglectus *+ *diana *+ *mona *group clade [7,14]. In the current study, several relationships within the genus are supported by *Alu *insertions that have statistical support. For example, the clustering of *C. ascanius *and *C. cephus *within the *cephus *group (represented in this study by *C. ascanius*; *C. cephus *and *C. petaurista*) is well supported. The close relationship between the *mitis *group (represented here by *C. nictitans*) and the *cephus *group is also strongly supported. The *diana *group (represented here by *C. diana*) appears to be basal within the arboreal *Cercopithecus *clade. However, this hypothesis deserves further investigation, owing to the relatively low level of support for the placement of *C. wolfi*. Overall, these results are in good agreement with previous studies using X and Y chromosome DNA data, and provide robust independent evidence for the suggested relationships [7,14].

### Arboreal to terrestrial lifestyle transition

Our results provide strong support for a single evolutionary transition from arboreality to terrestriality within the tribe Cercopithecini. The inference of a single transition to a ground-dwelling lifestyle suggests that such changes in substrate preference are probably rare in the history of the primates and therefore may lend greater significance to a similar change at the beginning of the hominin radiation. In addition, the guenon transition to a terrestrial lifestyle is estimated to have occurred at the Miocene/Pliocene boundary [7]. This is significant because the same time window brackets the origins of hominin terrestrial bipedalism. Thus, similar factors may have driven early human ancestors and the terrestrial guenon progenitor to a largely ground-dwelling way of life.

### Incongruent patterns of *Alu *insertions among lineages

There are three major scenarios in SINE-based phylogenetic analyses that can lead to confounding results: adjacent independent insertions, incomplete lineage sorting, and hybridization between species. Adjacent independent insertions refer to the scenario that in different species, different *Alu *elements are inserted in close genomic proximity and are therefore amplified within the same PCR amplicon during genotyping. In this study, we sequenced all informative loci to confirm that the appropriately size amplicon contained the same *Alu *insertions, and all loci containing parallel independent insertions have been excluded or treated as independent markers (see Results section). Therefore, the incongruence observed in our current dataset cannot be explained by invoking this explanation.

Incomplete lineage sorting is caused by alternative fixation/elimination of ancestral polymorphisms in populations of daughter lineages. Observations of incomplete lineage sorting can be particularly problematic when examining taxa that have undergone rapid bursts of speciation [22,34]. It is believed that the tribe Cercopithecini diverged from tribe Papionini about 11 million years ago [7,35]. However, owing to the lack of fossil evidence, the divergence times of subsequent groups are less certain. For the arboreal *Cercopithecus *clade, multiple speciation events may have occurred during a very short period to create the six species groups, containing the >20 species we see today [1]. If that is the case, many polymorphic *Alu *elements present in the guenon ancestral population have the potential to remain polymorphic before, during, and after these speciation events. These insertions were eventually fixed in or lost from the genomes of daughter species, and may have resulted in the incongruent insertion presence/absence patterns observed.

Finally, occasional introgressions via hybridization present yet another hypothesis to explain the incongruent *Alu *patterns. The ranges of closely related species within many guenon species groups often overlap geographically, and many of these species are known to hybridize with each other. For example, it is known that hybridizations occur between the red-tailed monkey group (*cephus *species group) and the blue monkey group (*mitis *species group, represented here by *C. nictitans*) in their contact zones [36]. These hybridizations may have contributed to the polytomy of *C. ascanius*, *C. cephus*, *C. petaurista *(the *cephus *species group) and *C. nictitans *(the *mitis *species group) in our study. In fact, introgression may even have introduced certain *Alu *insertion loci into species other than the arboreal *Cercopithecus *clade and provide a plausible explanation for some of the incongruent results in our study.

## Conclusion

Based on 179 *Alu *insertions, a robust phylogenetic hypothesis has been constructed and the placement for *Miopithecus *is inferred with statistically significant support for the first time. In addition, our observations indicate that the group of arboreal *Cercopithecus *species may have experienced a series of rapid, even simultaneous, speciation events, and thus a number of *Alu *insertions have been randomly fixed in different lineages. Hybridization between different species in the tribe may have also contributed to the complex patterns in our dataset. Owing to the unidirectional insertion property and extremely low removal rate of fixed SINE insertions, these markers can be used to construct phylogenetic hypothesis with high statistical power and provide evidence independent to sequence-based molecular phylogenetic studies.

## Methods

### Computational data mining

Genomic sequences from *Chlorocebus aethiops*(African green monkey) were obtained from the NIH Intramural Sequencing Center [37], as part of the Comparative Vertebrate Sequencing Initiative. The sequences were broken into 10000-bp fragments and compared with the human genome (hg18) and the rhesus monkey genome (rheMac2) using the BLAST-Like Alignment Tool (BLAT) [38]. Fragments containing insertion/deletions were extracted and annotated to identify putative lineage-specific *Alu *insertions using previously reported methods [39]. In order to allow a focus on the cercopithecine lineage, only *Alu *elements present in the *Chlorocebus aethiops *sequence but absent in the orthologous regions of the rhesus monkey genome were excised along with 1000 bp of flanking sequence in both directions. Flanking oligonucleotide primers for PCR amplification of each *Alu *element were then designed using Primer3 software [40]. The primers were subsequently screened against the GenBank NR database using the Basic Local Alignment Search Tool (BLAST) program [41] to determine if they resided in unique DNA sequences.

### PCR display methodology and PCR genotyping

The *Alu *element PCR-display methodology and *Alu*-specific primers described by Xing *et al *[28] were used for identifying species-specific *Alu *insertions. To prevent ascertainment bias, every cercopithecine species in this study was subject to this methodology and *Alu *insertion loci were identified from all species. Oligonucleotide primer pairs were initially tested using *Chlorocebus aethiops *DNA templates with a temperature gradient PCR (48–60°C) to determine the most appropriate annealing temperature for further analysis. All loci were screened against a primate panel that was composed of DNA samples from 12 Old World primate species (Table 1). Because the quantity of genomic DNA samples for most species is limited, all samples except *Chlorocebus aethiops *were subjected to whole genome amplification using the GenomiPhi genome amplification kit (Amersham, Sunnyvale, CA, USA) following the manufacturer's instructions. The amplified samples were then purified and divided into aliquots for locus-specific PCR analysis.

PCR amplification of each locus was performed as described by Xing *et al *[28]. The resulting PCR products were run on 2% agarose gels with 0.25 μg of ethidium bromide and visualized using ultraviolet fluorescence. Detailed information for each locus including primer sequences, annealing temperature, PCR product sizes, chromosomal locations and amplification results are available on our website (supplemental table. located under "Publications") [42].

### DNA sequence analysis

For the potentially informative loci, all PCR amplicons that appeared to have *Alu *insertions were subjected to sequence analysis to verify the presence of the *Alu *elements. Selected taxa from several uninformative loci were also sequenced to confirm the presence of the *Alu *elements. Individual PCR products were either directly sequenced or were cloned and sequenced as described previously [29]. Sequences for each locus were aligned against rhesus monkey (when available) or human orthologous sequence obtained via the BLAT search. Sequence alignments of these loci are available from our website (supplemental table. located under "Publications") [42]. The DNA sequences generated for this project have been deposited in GenBank (accession numbers DQ977747-DQ978210).

### Phylogenetic analysis

*Alu *insertion loci were included in phylogenetic analysis if amplicons were generated in at least seven out of the 11 cercopithecine taxa in our panel, and only 2 distinct classes of amplicons were obtained (i.e. *Alu *filled size and pre-integration or *Alu *empty size). Any primer pair that generated multiple paralogous fragments across the panel was excluded from the analysis.

We implemented an exhaustive search in PAUP* software (version 4.0b10) [43] using Dollo parsimony and designating the *Pygathrix nemaeus *as an outgroup taxon. Presence of the insert was coded as "1" and absence of the insert as "0". If no amplification was observed for a given locus in any taxon, the character state was coded as unknown ("?"). For loci containing adjacent independent insertion events, the independent insertions were treated as independent markers. In total, 10000 bootstrap replicates were performed on the data. A statistical test for evaluating SINE insertions based on a likelihood model [32] was also performed to assess the statistical significance of each branch of the resulting tree.

## Authors' contributions

JX, AJT, TRD and MAB designed the study. JX, HW, YZ and DAR performed the experiments. JX and HW analyzed the dataset. MAB directed the project. All authors were involved in preparing the manuscript.

## Figures and Tables

**Table 1:** DNA samples of all species examined in this study.

**Species Names**	**Common Names**	**Origin**	**ID number**
*Allenopithecus nigroviridis*	Allen's swamp monkey	IPBIR*	PR00198
*Cercopithecus ascanius*	Red-tailed monkey	IPBIR*	PR00566
*Cercopithecus cephus*	Mustached guenon	IPBIR*	PR00531
*Cercopithecus diana*	Diana monkey	Tosi†	N/A
*Cercopithecus lhoesti*	L'Hoest's monkey	IPBIR*	PR00283
*Cercopithecus nictitans*	White-nosed guenon	Tosi†	N/A
*Cercopithecus petaurista*	Lesser white-nosed monkey	IPBIR*	PR00762
*Cercopithecus wolfi wolfi*	Wolf's guenon	IPBIR*	PR00486
*Chlorocebus aethiops*	African green monkey	ATCC‡	CCL70
*Erythrocebus patas*	Patas monkey	SDFZ§	KB5435
*Miopithecus talapoin*	Talapoin	SDFZ§	OR 755
*Pygathrix nemaeus*	Red-shanked Douc langur	SDFZ§	OR 259

* Integrated Primate Biomaterials and Information Resource, .

† Blood sample from Dr. Anthony Tosi.

‡ Cell line provided by the American Type Culture Collection.

§ Frozen Zoo^®^, San Diego Zoo, .
